# Medical Complexity, Mortality Among High-Cost Medicare Advantage Enrollees: Palliative, Hospice Implications

**DOI:** 10.1093/geroni/igab046.3217

**Published:** 2021-12-17

**Authors:** Samuel Amodeo, Henrik Kowalkowski, Lauren Bangerter, Halley Brantley, David Cook, Nicholas Jones

**Affiliations:** 1 OptumLabs at UnitedHealth Group, Inver Grove Heights, Minnesota, United States; 2 UnitedHealth Group, Minneapolis, Minnesota, United States; 3 OptumLabs, Eden Prairie, Minnesota, United States; 4 UHG, Minnetonka, Minnesota, United States; 5 United Health Group, Optum Labs, Minnesota, United States; 6 Brown University School of Public Health, Brown University, Rhode Island, United States

## Abstract

Older adults with high medical spend require tailored interventions and care delivery to meet their complex needs. Palliative is a high-value solution for high-cost patients because it provides relief from the symptoms, pain, and stress associated with multiple conditions. Likewise, other high-cost patients may be closer to end-of-life and therefore benefit from hospice care. For Accountable Care Organizations (ACOs) and hospitals to implement palliative care, these programs must identify and target the high-need patient populations. This study explored patterns of spending and mortality across 4 years (2016-2019) using claims from 1,701,647 patients continuously enrolled in UnitedHealth Group Medicare Advantage (mean age=73.7; S.E.=0.01). Patients with healthcare spend in the top decile were segmented into three subgroups based on health conditions and spend patterns. Analyses identified a subgroup of patients (mean age=76.6; S.E.=0.04), with the highest rate of mortality, and significantly more chronic conditions and frailty, indicating their cost and mortality was driven by medical complexity. Odds ratios from a multinomial logistic model tie blood formulation drugs (OR XX), medicative procedures (OR XX), and nonhospital-based care (OR XX) to members of this subgroup may be connected to short-term mortality. There is a critical need to identify patients who stand to benefit from palliative and end of life care, this is particularly true for high-cost high-need patients. Our study suggests that patterns of medical complexity and morality within high-cost patient subpopulations can be used to identify high-cost patients who would benefit from palliative or hospice care.

